# Impending perforation near ileocecal junction due to phytobezoar impaction and intraluminal polyp: a case report

**DOI:** 10.1186/s13256-022-03356-0

**Published:** 2022-03-30

**Authors:** Mehwish Mooghal, Asrar Ahmad, Adnan Safi, Wajiha Khan, Naveed Ahmad

**Affiliations:** 1 Department of Surgery, Bahria University Medical and Dental College, Karachi, Pakistan; 2 Department of Surgery, PNS Shifa Hospital, Karachi, Pakistan; 3 Department of Medicine and Surgery, Nishtar Medical University and Hospital, Nishtar Road, Multan, 60000 Pakistan; 4 Dow University of Health and Sciences, Karachi, Pakistan

**Keywords:** Bezoar, Intestinal obstruction, Intraluminal polyp, Computed tomography, Phytobezoars, Phytobezoar impaction

## Abstract

**Introduction:**

Bezoars and polyps are an uncommon cause of mechanical intestinal obstruction. There are four different kinds of bezoars: phytobezoars, made of vegetables and fibers; trichobezoars, resulting from the ingestion of hair and frequently an expression of psychiatric disorders; lactobezoars, which are formed of milk curd; and pharmacobezoars, caused by drugs and medications. Signs and symptoms classically vary from abdominal pain to constipation, nausea, vomiting, and abdominal distension. We present a rare case of impending perforation along with an intraluminal polyp near ileocecal junction due to phytobezoar impaction.

**Case presentation:**

Our patient was a 59-year-old Sindhi female with a known history of interstitial lung disease and hypertension who presented to the emergency department with complaints of abdominal pain and constipation for 1 week, vomiting for 5 days, and abdominal distension for 2 days. After a preoperative examination and her failure to respond to conservative therapy, she was taken to the operating room for exploratory laparotomy. A hard intraluminal mass was suspected to be obstructing the small bowel at the site of impending perforation. This mass was a phytobezoar along with an intraluminal polyp. Resection of the affected segment was performed, followed by ileoileal anastomosis, and a drain was left. The patient was discharged 1 week later and was found to be well with no complaints at 3 weeks follow-up.

**Conclusions:**

Early diagnosis of bezoars is important for early intervention and prevention of complications. Our case is unique as phytobezoar with intraluminal polyp is a rare clinical finding. Moreover, the signs and symptoms with which the patient presented are nonspecific and can be seen with multiple surgical emergencies.

## Introduction

Bezoars consists of concretions of poorly or undigested material within the gastrointestinal (GI) tract, most commonly in the stomach. Phytobezoars composed of vegetables and/or fruit matter are common. Other varieties are trichobezoars (hair), pharmacobezoars (medications), lactobezoars (milk proteins in milk-fed babies), and so on [1].

The main components of phytobezoars are cellulose or hemicellulose, tannin, and/or lignin. Factors that promote phytobezoar formation are previous gastric surgery (most common), excessive high-fiber diet consumption, medications that delay gastric motility, rapid swallowing of large amounts of foods, poor mastication due to artificial dentures (mainly in the elderly) hypothyroidism, diabetes mellitus, renal failure, or postoperative adhesions [2].

Polyps are rare benign mesenchymal tumors arising from the mucosa and submucosa of the GI tract. Risk factors for polyp include trauma, allergy, and genetic factors as well as bacterial, physical, chemical, and metabolic stimuli that would eventually lead to the development of polyps. Phytobezoars and polyps most commonly present with abdominal discomfort, fullness or pain, difficulty in swallowing, or anorexia. Furthermore, bezoar-related intraluminal pressure results in mucosal ulceration and necrosis, which can present with symptoms related to GI bleeding, such as anemia, bloody stools, hematemesis, and so on [3]. Very rarely, phytobezoars along with intraluminal polyp involving the small intestine present as acute abdomen, vomiting, abdominal distension, or hypotension, due to intestinal obstruction or perforation [4].

Here we present a case of impending perforation due to phytobezoar impaction and an intraluminal polyp near ileocecal junction in a patient with no previous history of any GI pathology or surgery.

## Case report

A 59-year-old Sindhi female patient, with known interstitial lung disease (ILD) and hypertension, presented to the emergency department with complaints of abdominal pain and constipation for 1 week, vomiting for 5 days, and abdominal distension for 2 days. Abdominal pain was centrally located, gradual in onset, continuous, dull in character, severe in intensity, nonradiating, and associated with seven episodes of bilious vomiting and constipation. The patient passed no flatus and stool for 1 week. Past medical history consisted of hospital admission 7 years before presentation because of uncontrolled hypertension that caused her to become unconscious and receive ventilatory support.

On examination, the patient was hemodynamically stable with pulse rate 110 beats per minute, blood pressure 140/90 mmHg, temperature 98°F, respiratory rate 20 breaths per minute, and O_2_ saturation 93% at 5 l O_2_. Her abdomen was distended with generalized tenderness, guarding, dull percussion notes, and sluggish bowel sounds. Chest was bilaterally audible with crackles in whole lung field.

**Fig. 1:**
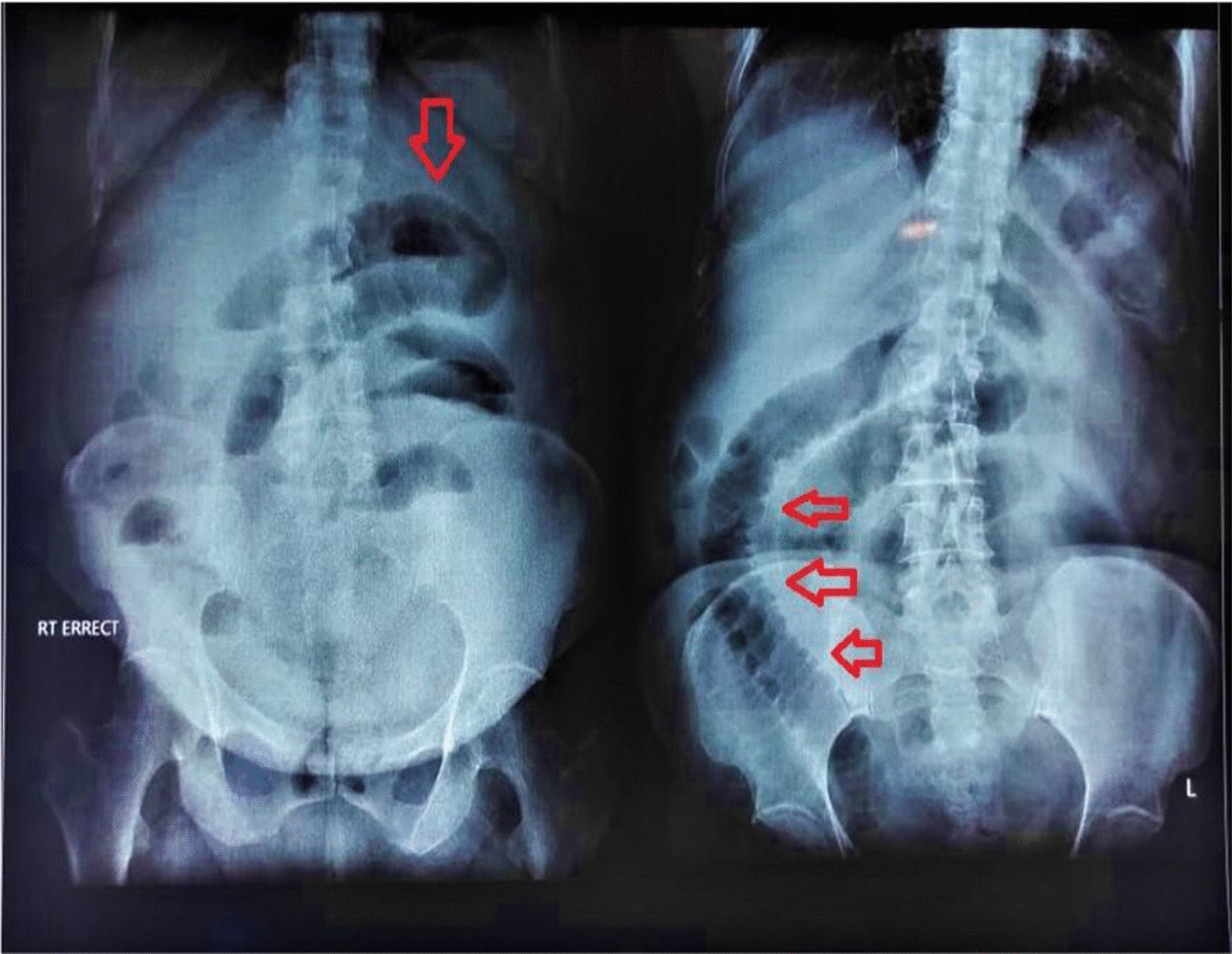
Erect x-ray abdomen showing valvulae conniventes and featureless ileum and dilated small bowel loops. Red arrows shows valvulae conniventes and featureless ileum and dilated small bowel loops

**Fig. 2:**
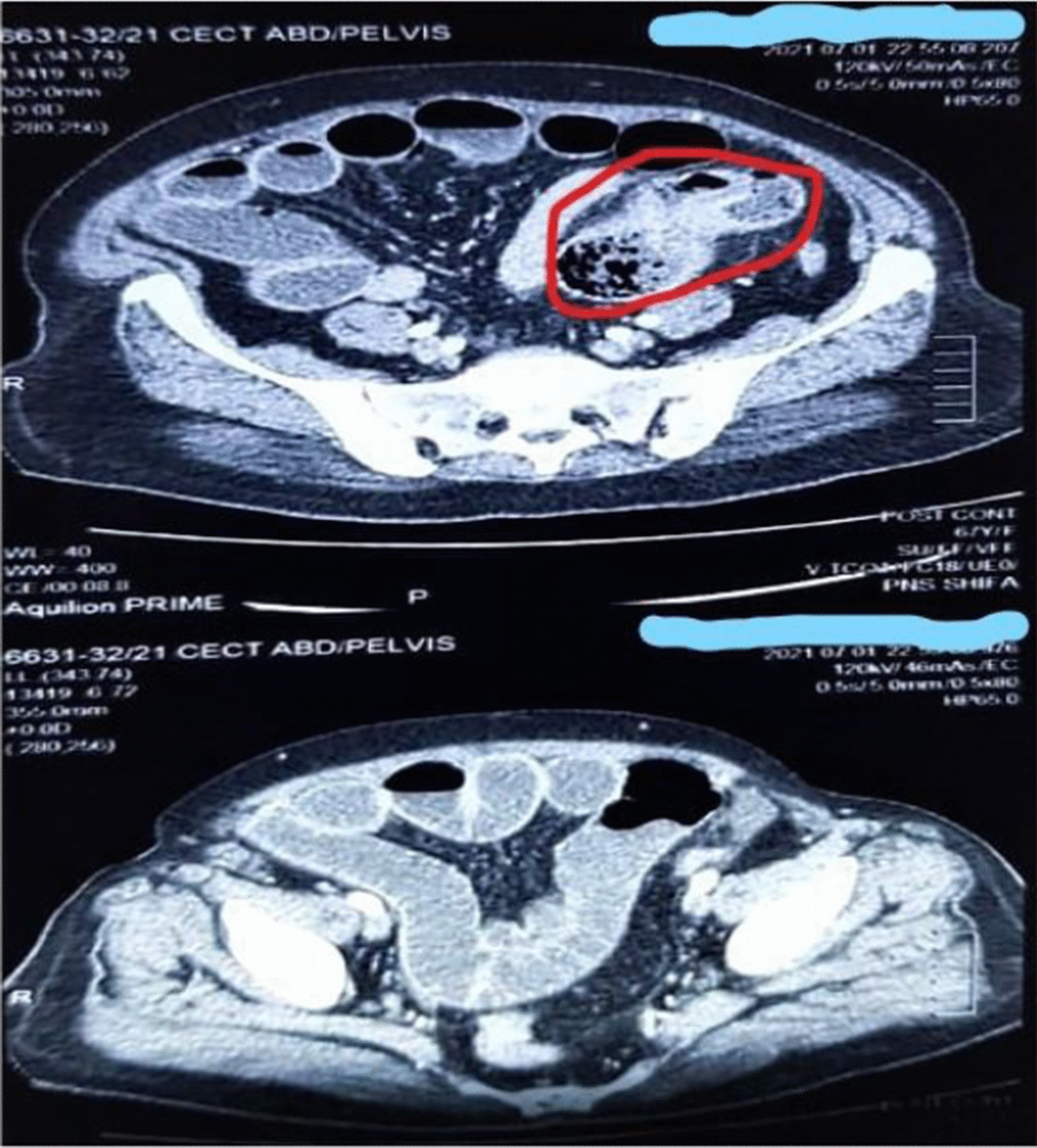
CECT showing dilated small bowel loops with air fluid levels and intraluminal mass. Red circle shows air fluid levels. Blue lines are actually hiding the identity in of the patient

Blood investigations showed elevated total leukocyte count (TLC) with neutrophils 84%, serum ferritin level of 366.9 ng/ml, erythrocyte sedimentation rate (ESR) 41 mm per hour, and carcinoembryonic antigen (CEA) 3.24 ng/ml. Radiological investigations including chest x-ray (CXR), x-ray abdomen (AXR), and contrast-enhanced CT (CECT) were requested and performed, which suggested the clinical diagnosis of subacute small bowel obstruction (Figs. 1, 2).

**Fig. 3:**
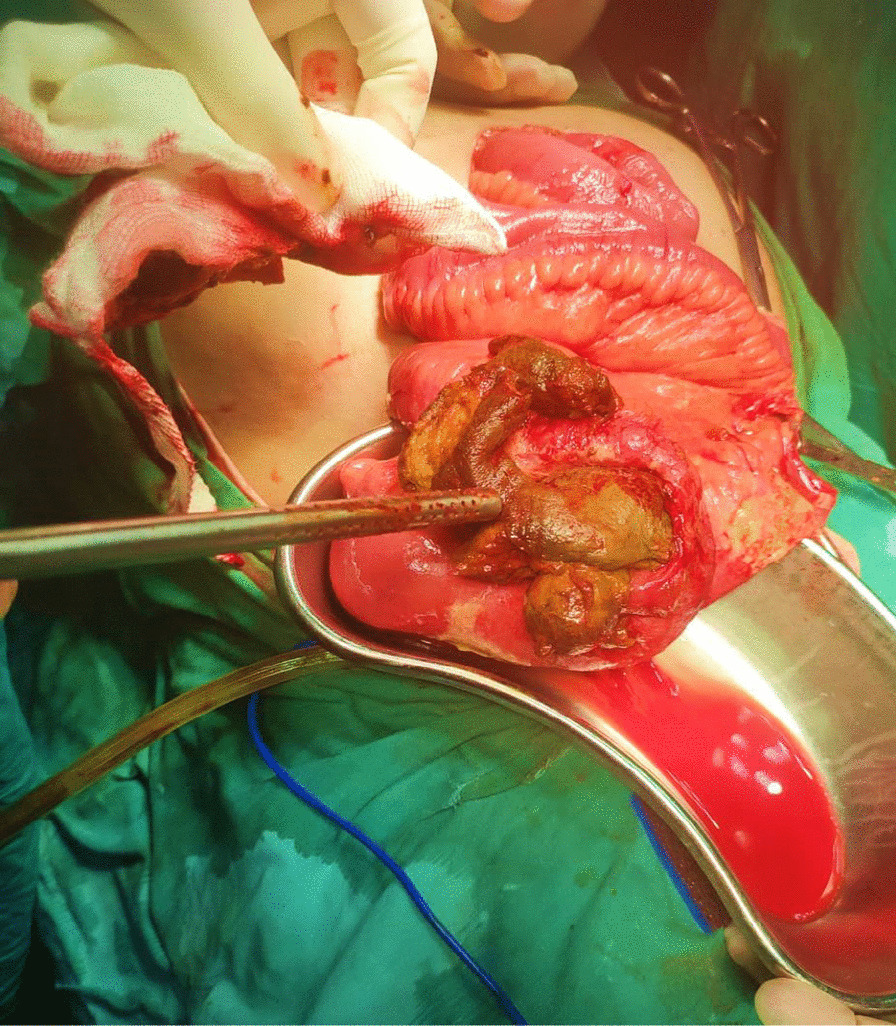
Specimen showing phytobezoar

**Fig. 4:**
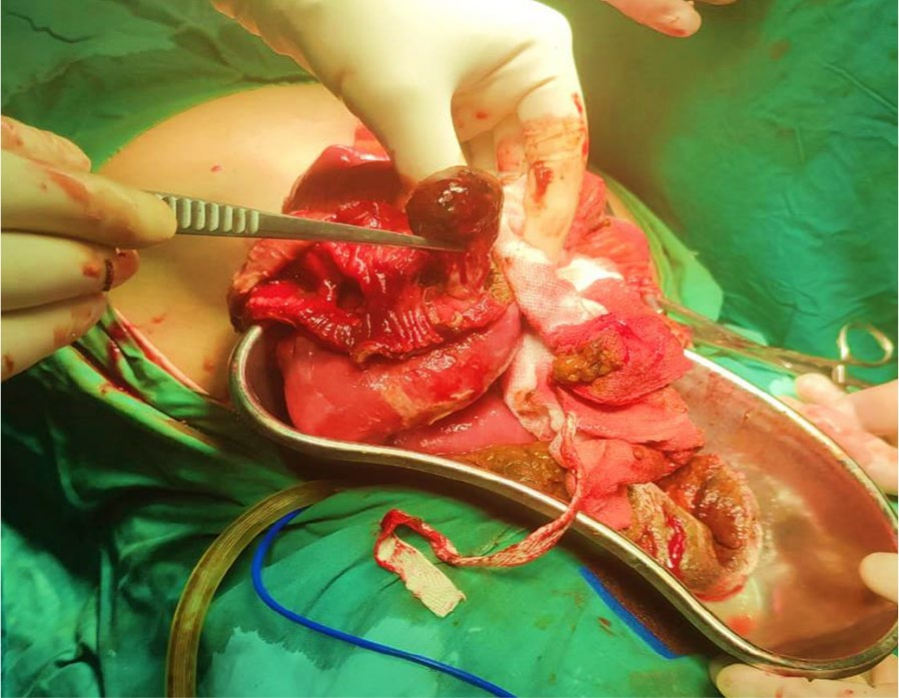
Specimen showing an intraluminal polyp

An exploratory laparotomy was performed through a midline incision showing impending perforation, with petechiae on the wall of terminal ileum, 15 cm proximal to ileocecal valve. A hard intraluminal mass was suspected to be obstructing the small bowel at the site of impending perforation. The gut was cut opened at this location from antimesenteric border and a 5 cm phytobezoar along with a polyp was found (Figs. 3, 4). Resection of the affected segment was performed, which was then followed by ileoileal anastomosis, and a drain was left.

The patient was discharged 1 week later, and was found to be well with no complaints at 3 weeks follow-up.

## Discussion

Bezoars are concretions of poorly digested fibers, skins, and seeds of fruits and vegetables or foreign bodies in the alimentary tract. They usually form in the stomach, but they can migrate to the small bowel, where they may cause obstruction. There are four types of bezoars. Phytobezoars are the most common, composed of vegetable matter and containing a large amount of nondigestible fibers such as cellulose, hemicellulose, and fruit tannins. Trichobezoars are gastric concretion of hair fibers, and usually present in patients with history of psychiatric issues and in children with mental retardation. Pharmacobezoars consist of medication bezoars, which adhere in bulk, such as cholestyramine and antacids. Lactobezoars are milk curd secondary to infant formula, described in low-birth-weight neonates fed highly concentrated formula within the first week of life [5].

Phytobezoars along with intraluminal polyp usually present with abdominal pain, epigastric distress, nausea and vomiting, and small bowel obstruction (SBO) as the main clinical symptoms. Feeling of fullness or bloating, dysphasia, anorexia with weight loss, and even gastrointestinal hemorrhage could also be seen [6]. Diagnosing intraluminal polyp with phytobezoar-induced small bowel obstruction can be challenging preoperatively. Abdominal radiography can highlight air–fluid levels associated with mechanical obstruction. However, many authors agree that it is nonspecific [2]. The diagnostic rate of abdominal ultrasound in detecting phytobezoars is reported to be 88–93% [7]. In this patient, x-ray abdomen showed features of small bowel dilation with apparent valvulae conniventes indicating small bowel obstruction. A barium enema or endoscopy can also be used, although their diagnostic accuracy is limited [8]. CT with contrast enhancement has a sensitivity of 90% and a specificity of 57% in recognizing bezoars [2], and is now the gold standard in the diagnosis of bezoars and SBO. It permits the differential diagnosis of other bowel masses and identifies signs such as ascites, wall bowel thickening, proximal lumen dilatation, and intestinal infarction. CT can aid in choosing a conservative or a surgical/endoscopic treatment strategy; in most cases of bezoars and SBO, it leads to targeted surgical therapy [2].

The treatment of choice for SBO due to bezoar along with polyp in intestinal lumen is surgery. In most cases, the impaction of bezoar takes place in the narrowest segment of the small bowel, which is located 50–75 cm from the ileocecal valve [9]; because it is narrow, slow intestinal motility and a large amount of water absorption harden the bezoar, resulting in its loss of motility. In this case, hard intraluminal mass around 5 cm in length and 20 cm proximal to ileocecal junction was found obstructing the lumen of small intestine with pressure necrosis of the lumen wall at the site of obstruction by phytobezoar. Enterotomy is the procedure to remove such obstruction. If it is in the form of a hard mass like in this patient, laparotomy and segmental bowel resection need to be performed, followed by ileoileal anastomosis. During surgery, thorough exploration of the abdominal cavity should be done to exclude the presence of concomitant gastric bezoar or intestinal bezoars as well as any intraluminal polyp. Laparoscopic management of phytobezoar-induced small bowel obstruction has been reported; however, this requires expertise. Recurrence is common unless the underlying predisposing condition is corrected.

However, the best way to prevent phytobezoar is through good eating habits, high-fiber diet particularly in patients with gastric surgery, and introduction of prophylactic medication, in high-risk groups and those with previous history, to improve gastric motility and psychiatric follow-up in patients with psychiatric disease.

## Conclusion

Bezoar along with intraluminal polyp-induced small bowel obstruction is a very rare entity that may be difficult to establish diagnosis preoperatively. There should be high index of suspicion in patients with signs and symptoms of small bowel obstruction. The presence of well-defined intraluminal mass with mottled gas pattern at the site of obstruction in CT abdomen raises the suggestion of bezoar. Surgery is the best management of bezoar-induced small bowel obstruction. Diet modification and management of underlying disorders are the best prevention.

## Data Availability

Not applicable.
